
JOURNAL INFORMATION
==============================
NLM Title Abbreviation: ScientificWorldJournal
Journal ID: ScientificWorldJournal
NLM Journal ID: 101131163
Journal ID: TSWJ

The Scientific World Journal

ISSN: 2356-6140
EISSN: 1537-744X
Publisher: Wiley

ARTICLE INFORMATION
==============================
PMCID: PMC5901205
PMID: 17619772
DOI: 10.1100/tsw.2007.158
Article ID: 618179
Article version: 1
Subjects: Editorial

Hope and Challenges in the Management of Advanced Renal Cell Carcinoma

Clark Peter E. *
Vanderbilt University Medical Center, A-1302 Medical Center North, Nashville, TN 37232-2765, USA
*Peter E. Clark: peter.clark@vanderbilt.eduAcademic Editor: Peter E. Clark

Publication date: 2007
Electronic publication date: 2007 May 1
Volume: 7
First page: 869
Last page: 869
Received 2007 Apr 12; Accepted 2007 Apr 12
Copyright: Copyright © 2007 Peter Clark.
Copyright year: 2007
License: This is an open access article distributed under the Creative Commons Attribution License, which permits unrestricted use, distribution, and reproduction in any medium, provided the original work is properly cited.
License URL: https://creativecommons.org/licenses/by/3.0/

==============================
Editorial
Special Issue: Renal Cancer: The Treatment in Metastatic Disease
*TheScientificWorldJOURNAL* (2007) 7, 869
TSW Urology
ISSN 1537-744X; DOI 10.1100/tsw.2007.158

TheScientificWorldJOURNAL
www.thescientificworld.com

### Hope and Challenges in the Management of Advanced Renal Cell Carcinoma

Peter E. Clark

*Vanderbilt University Medical Center, A-1302 Medical Center North, Nashville, TN 37232-2765*

**E-mail:** peter.clark@Vanderbilt.Edu

*Received April 12, 2007; Accepted April 12, 2007; Published May 1, 2007*

---

The management of clinically localized renal cell carcinoma (RCC) has been grounded in surgical extirpation for many decades now, and remains the only consistent approach to offer a cure for patients with this disease. For many years, therapy for advanced disease had been frustrated by its resistance to conventional cytotoxic chemotherapy. Cytokine-based approaches formed the mainstay of our armamentarium and offered response rates on the order of 10–20%, with modest improvements in survival and long-term disease-free survival in less than 5%. Clearly, new approaches were needed. Within the last several years, new advances have begun to revolutionize the management of advanced RCC and offer hope where before the outlook was often bleak. These included a more accurate and proven role for aggressive surgical resection of the primary lesion and, in selected cases, metastectomy as well as refinements in cytokine-based therapy. In carefully selected patients, the possibility of allogeneic, hematopoietic, stem-cell transplantation has been explored. The ability to risk stratify patients accurately has substantially improved and current approaches to the molecular classification of renal neoplasms offer newfound opportunities, not only in predicting an individual person's prognosis, but also potential new targets for therapy. It is not too farfetched to envision, in the not-so-distant future, individualized, targeted therapy based on the molecular defects in an individual's particular tumor. Perhaps the most profound change, however, has come out of the work on the molecular biology of RCC. In particular, the elucidation of the central role played by aberrations of the von Hippel Lindau gene/protein and its downstream consequences in clear cell (conventional) carcinoma. These insights have led to a series of exciting new approaches, so-called "Targeted Therapy", that have revolutionized how we approach advanced RCC. The articles in this special issue will address many of these changing paradigms in the management of RCC, and highlight how far we have come and how far we still need to go. The war against RCC has not been won yet, but as a research and therapeutic community, we are starting to win some of the smaller battles, one at a time. The key to future progress depends on an ongoing commitment to enrollment in carefully designed and executed clinical trials, the courage of the patients who participate in them, and continued dedication on the part of clinicians and scientists who work on this difficult and challenging disease.

---

**This article should be cited as follows:**

Clark, Peter E. (2007) Hope and challenges in the management of advanced renal cell carcinoma. *TSW Urology* **2,** 143. DOI 10.1100/tswurol.2007.121.

---

©2007 with author.
Published by TheScientificWorld; www.thescientificworld.com
